# Lung microbiome and cytokine profiles in different disease states of COPD: a cohort study

**DOI:** 10.1038/s41598-023-32901-0

**Published:** 2023-04-07

**Authors:** Qing Xue, Yu Xie, Yukun He, Yan Yu, Guiju Fang, Wenyi Yu, Jianhui Wu, Jiwei Li, Lili Zhao, Xinyu Deng, Ran Li, Fang Wang, Yali Zheng, Zhancheng Gao

**Affiliations:** 1 The Third Clinical Medical College, Fujian Medical University, Ningde Municipal Hospital, Ningde, Fujian China; 2 Department of Respiratory and Critical Care Medicine, Peking University People’s Hospital, Beijing, 100044 China; 3 Department of Respiratory Medicine, Shanghai Ninth People’s Hospital, Shanghai Jiao Tong University School of Medicine, Shanghai, China; 4 Department of Respiratory, Critical Care, and Sleep Medicine, Xiang’an Hospital of Xiamen University, School of Medicine, Xiamen University, Xiamen, 361101 China

**Keywords:** Bacteria, Microbial communities

## Abstract

Increasing evidence indicates that respiratory tract microecological disorders may play a role in the pathogenesis of chronic obstructive pulmonary disease (COPD). Understanding the composition of the respiratory microbiome in COPD and its relevance to respiratory immunity will help develop microbiome-based diagnostic and therapeutic approaches. One hundred longitudinal sputum samples from 35 subjects with acute exacerbation of COPD (AECOPD) were analysed for respiratory bacterial microbiome using 16S ribosomal RNA amplicon sequencing technology, and the sputum supernatant was analysed for 12 cytokines using a Luminex liquid suspension chip. Unsupervised hierarchical clustering was employed to evaluate the existence of distinct microbial clusters. In AECOPD, the respiratory microbial diversity decreased, and the community composition changed significantly. The abundances of *Haemophilus*, *Moraxella*, *Klebsiella*, and *Pseudomonas* increased significantly. Significant positive correlations between the abundance of *Pseudomonas* and TNF-α, abundance of *Klebsiella* and the percentage of eosinophils were observed. Furthermore, COPD can be divided into four clusters based on the respiratory microbiome. AECOPD-related cluster was characterized by the enrichment of *Pseudomonas* and *Haemophilus* and a high level of TNF-α. *Lactobacillus* and *Veillonella* are enriched in therapy-related phenotypes and may play potential probiotic roles. There are two inflammatory endotypes in the stable state: *Gemella* is associated with the Th2 inflammatory endotypes, whereas *Prevotella* is associated with the Th17 inflammatory endotypes. Nevertheless, no differences in clinical manifestations were found between these two endotypes. The sputum microbiome is associated with the disease status of COPD, allowing us to distinguish different inflammatory endotypes. Targeted anti-inflammatory and anti-infective therapies may improve the long-term prognosis of COPD.

## Introduction

Chronic obstructive pulmonary disease (COPD) is a chronic airway inflammation characterised by progressive and non-reversible airflow limitation and recurrent episodes of exacerbations, causing significant mortality worldwide^1^. Acute exacerbations of COPD (AECOPD), mostly induced by respiratory infections, are heterogeneous and vary in severity and phenotype^2^.

With the development of culture-independent methods for microbial genome sequencing, the microbiome compositions of the airways of patients with COPD have been characterised, both in stable states and during exacerbations^3^. Studies showed that the airway microbiome differs during exacerbations from that of the stable state and that these changes are associated with exacerbation phenotype, severity, and prognosis^4^. The patterns of inflammation observed in COPD are known as inflammatory endotypes^5^, and identifying these endotypes may allow for better therapeutic applications^6^. There are different microbiome characteristics among the inflammatory endotypes^4,7^.

Furthermore, host-microbiome interactions have attracted increasing attention. as they are helpful for better understanding the roles of host and airway microbiota during COPD exacerbations. There may be a synergistic response between the host and microbiome in COPD^8^. Some species are associated with airway inflammatory cells and mediators; a *Haemophilus*-predominant community is related to neutrophilic inflammation and elevated sputum IL-1β and TNF-α; meanwhile *Moraxella* is related to Th1 pathways, such as interferon signalling^3,9,10^.

However, most studies have compared exacerbation and stable state or explored the differences between diseased and healthy groups. The changes in the microbial community and cytokine profiles during COPD remain unclear. Therefore, we investigated longitudinal changes in the airway microbiome and cytokine levels of patients with different clinical states, including post-therapy and stability periods at follow-up.

## Materials and methods

### Study population

The study consecutively enrolled 35 patients diagnosed with AECOPD and hospitalized in Ningde City Hospital from January 2017 to August 2017 (ClinicalTrials.gov ID, NCT03236480). The diagnosis and assessment of the severity of COPD and assessment of AECOPD were made according to the recommendations of the Global Initiative for Chronic Obstructive Lung Disease (GOLD) committee^11^. Stable state was defined as being 4 weeks free from an exacerbation and post therapy state was defined as an improvement in the patient's respiratory symptoms after hospitalization that allows the patient to stop treatment and be discharged. The exclusion criteria were antibiotic using before admission, and the presence of other respiratory diseases. Table 1 showed the clinical characteristics and medication of study subjects. The average age of the participants was 72.4 years old, and 97.1% were male. Among them, 6 participants had never smoked, 17 had currently smoked and 12 had quit. Respiratory failure occurred in 17 subjects during the acute exacerbation, 4 of whom received non-invasive ventilation.

**Table 1:** The clinical characteristics of study subjects.

	Study population (n = 35)
Age, years	72.4 ± 9.2
Male	34 (97.1%)
BMI, kg/m^2^	21.5 ± 2.4
Pack-years	34.0 ± 24.2
Current smokers	12 (34.3%)
GOLD stage	
1	1 (2.9%)
2	8 (22.9%)
3	12 (34.2%)
4	14 (40.0%)
Comorbidities	
Coronary heart disease	9 (25.7%)
Diabetes mellitus	2 (5.7%)
Hyperlipidemia	1 (2.9%)
Chronic kidney disease	0 (0.0%)
AE frequency in the last year	3.0 (1.0–4.0)
AE leading to hospitalization in the last year	1.0 (0.0–1.0)
Eosinophils, %	7.9 (1.2–18.0)
Eosinophils, × 10^9^/L	0.9 (0.2–3.0)
Only glucocorticoid use during acute exacerbation	5 (14.3%)
Only antibiotic use during acute exacerbation	6 (17.1%)
Both glucocorticoid and antibiotic use during acute exacerbation	24 (68.6%)
Bronchodilator/steroid combination preparation use during the stable period	19 (54.3%)
Inhaled bronchodilators use during the stable period	2 (5.7%)
Outcome	
Type I respiratory failure	7 (20.0%)
Type II respiratory failure	10 (28.6%)
Non-invasive ventilation	4 (11.4%)
Tracheal intubation	0 (0.0%)
In-hospital mortality	1 (2.9%)

*BMI* body mass index, *GOLD* global initiative for chronic obstructive lung disease, *AE* acute exacerbation.

Clinical data during hospitalization were collected using a standard electronic medical record. Follow-up for 120 days was conducted after discharged.

### Sample collection, sequencing, and cytokine measurements

Qualified spontaneous sputum samples based on the epithelial cell count were collected 24 h after admission prior to any systemic therapy, 24 h before discharge and 1, 2, and 3 months after discharge. We collected a total of 113 samples, including 35 in the acute exacerbation state, 35 in the post therapy state and 43 during follow up period. After strict filtering, a total of 100 sputum samples with high quality and sufficient amount of DNA for sequencing were collected, including 31 in the acute period provided by 31 patients, 33 in the post-therapy period provided by 33 patients, and 36 during the stable period provided by 17 patients. Detailed procedures of sputum specimens processing, DNA extraction, PCR, and library preparation were supplied in supplementary materials. The library was sequenced on an Illumina NovaSeq platform and 250 bp paired-end reads were generated. Specimen-free reagent for extraction and PCR amplification was used as negative controls. A total of 12 cytokines were measured in the sputum supernatants using Luminex Human Magnetic Assay Kit (LXSAHM-12; R&D Systems, Minneapolis, MN, USA). Detailed information has been described in an article previously published^12^.

### 16S rRNA gene sequencing analysis

Paired-end reads were truncated by cutting off the barcodes and primer sequences and merged using FLASH (-m 10 -M 213 -P 33 -r 219 -F 300) (Version 1.2.11, http://ccb.jhu.edu/software/FLASH/)^13^. Quality filtering were performed using the fastp (Version 0.20.0) software. Chimeras were removed using Vsearch (Version 2.15.0) to obtain the effective tags^14^. Then denoise was performed with DADA2 in the QIIME2 software^15^ to obtain initial Amplicon Sequence Variants (ASVs)^16^. A custom Naïve Bayes classifier was trained on the Greengenes 13_8 99% operational taxonomic units to assign taxonomy. Multiple sequence alignments were performed using QIIME2 software to study phylogenetic relationship of each ASV and the differences of the dominant species among different groups. ASVs with relative abundance ≥ 0.05% in at least three samples were filtered^17^ for subsequent analysis. Detailed methods of analysis of 16S rRNA gene sequencing and possible contaminations were supplied in supplementary material. All data are publicly available in the Sequence Read Archive (https://www.ncbi.nlm.nih.gov/sra) under accession number PRJNA861712.

### Ethics approval and consent to participate

Patients all gave written informed consent before their participation in the study. The Ethics Committee of Peking University People's Hospital approved the study (Approval number: 2016PHB202-01). This trial was conducted in accordance with the Declaration of Helsinki.

## Results

### Lung microbiome profiles

To characterise the bacterial community composition of pulmonary microbiota under various COPD states, 100 longitudinal sputum samples from 35 COPD patients during acute exacerbation, post-therapy, and stable periods were explored. In total, 13,884 ASVs were obtained. Figure S1 shows a sparse curve. The median frequency of feature sequences was 81735, the minimum frequency was 37377, and the maximum frequency was 131234. The minimum frequency was used as sampling depth to calculate core diversity, which was convenient for subsequent community diversity analysis.

**Figure 1:**
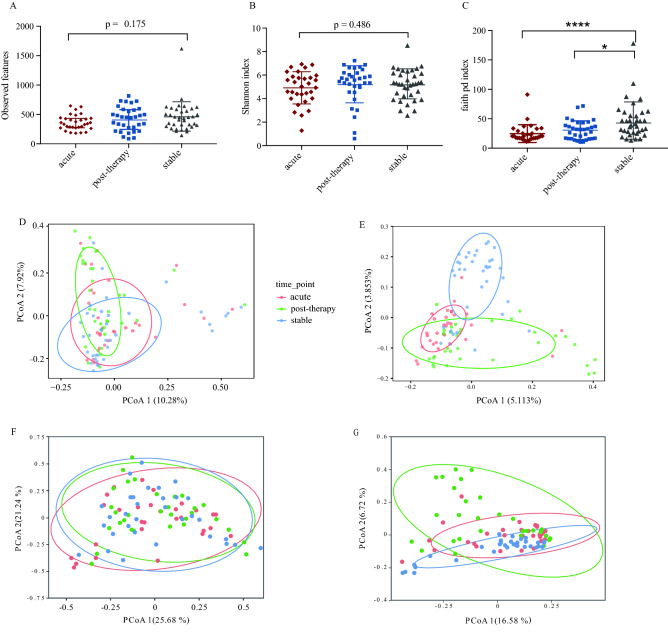
Diversity of respiratory microbial community in patients with different disease states. (**A**–**C**) The number of OTU observed, Shannon index and Faith PD index among the three groups. (**D**–**G**) The PCoA plots based on Bray Curtis, Jaccard distance, Weighted-Unifrac, and Unweighted-Unifrac distance. *OUT* operational taxonomic unit, *PCoA* principal coordinate analysis, *Faith PD* faith's phylogenetic diversity. *p < 0.05, ****p < 0.0001.

Based on a relative abundance of ≥ 0.05% in at least three samples^17^, 1275 ASVs were selected for community analysis. For alpha diversity, three indices, observed features, Shannon index, and Faith’s phylogenetic diversity (Faith PD) index for samples from the acute exacerbation period decreased and the Faith PD index showed a statistically significant difference compared to the that of the samples after treatment and in the stable period (p < 0.0001; Fig. 1A–C). The Adonis test based on four distance measurements, Bray–Curtis, Jaccard, Weighted-Unifrac, and Unweighted-Unifrac distances, revealed significant differences in respiratory microbial composition among the three disease states (p = 0.04, 0.001, 0.001, 0.001, respectively) (Table S1). Similarly, principal coordinate analysis (PCoA), based on these four distance measurements, showed that the three groups were clustered and well distinguished (Fig. 1D–G). These results indicate that the microbial communities of the disease states differed significantly, and diversity decreased during acute exacerbation. In addition, we compared the microbial composition of the different treatment groups (patients received antibiotics, steroids or both during exacerbation) as well as groups with different disease severity in the acute exacerbation and found no significant differences of alpha or beta diversity between the groups.

### Microbiome alterations during different status

**Figure 2:**
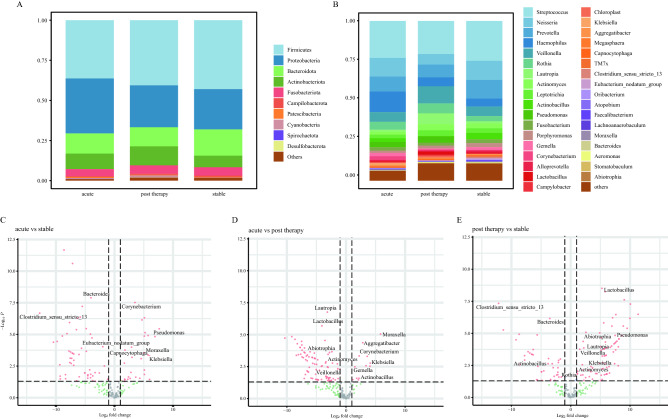
The distribution of microbiome in different disease states. Histogram of the relative abundance of the top 10 phyla (**A**) and the top 35 genera (**B**). (**C**–**E**) The volcanic plots of different taxa in three periods. Fold change is shown on the x axis and -log10 P (FDR corrected) on the y axis. Each dot in the diagram represents a sample. The red dots represent *p* < 0.05, | logFC |> 1; Green dots represent *p* > 0.05, | logFC |> 1; Blue dots represent *p* < 0.05, | logFC |< 1; Gray dot represents *p* > 0.05, | logFC |< 1. The black boxes represent the taxa that belong to top 35 genera.

The predominant phyla in the microbiome were Firmicutes (40.1%), Proteobacteria (28.3%), and Bacteroidetes (13.7%). The most abundant genera were *Streptococcus* (23.0%), *Neisseria* (10.1%), *Prevotella* (9.8%), *Haemophilus* (7.6%), and *Veillonella* (7.6%). Figure 2A,B show the top 10 phyla and top 35 genera across the different disease periods, respectively. Differential taxon analysis was performed at the genus level using the limma package in R (adjusted *p* < 0.05, | logFC |> 1). Twenty-four genera were enriched during acute exacerbation with the most dominant genera being *Haemophilus*, *Pseudomonas*, *Corynebacterium*, *Klebsiella*, *Capnocytophaga*, and *Moraxella*; In contrast, 47 genera decreased in abundance during acute exacerbation, including *Clostridium_sensu_stricto_13*, *Eubacterium_nodatum*, and *Bacteroides*. Notably, some taxa enriched in the stable status were not detected during the acute exacerbation, suggesting that the respiratory community was characterised not only by the increase in pathogenic bacteria during exacerbation but also by the decrease in colonising bacteria. Figure 2C–E shows the major differential taxa, and detailed results are provided in Tables S3–S5.

**Figure 3:**
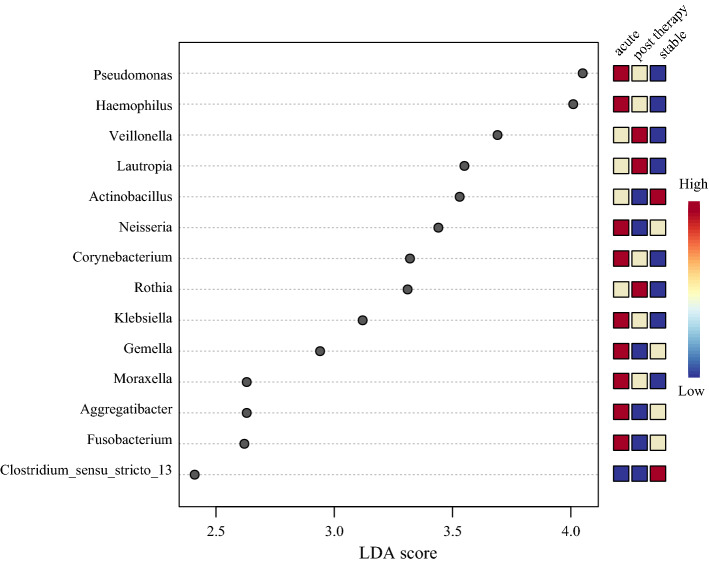
Histogram of LDA values of LEfSe analysis of respiratory microbiome with different disease status (*p* < 0.05 by the Wilcoxon test). *LDA* linear discriminant analysis, *LEfSe* linear discriminant analysis Effect Size.

Linear discriminant analysis effect size (LEfSe) analysis showed 14 discriminating genera among the three disease states (Table S6). *Haemophilus*, *Moraxella*, *Klebsiella*, *Pseudomonas*, *Neisseria, Corynebacterium, Gemella*, *Aggregatibacter*, and *Fusobacterium* were predominant in the acute exacerbation group; *Rothia*, *Veillonella*, and *Lautropia* were predominant in the post-therapy group; *Actinobacillus* and *Clostridium_sensu_stricto_13* were predominant during the stable period (Fig. 3).

### Association between genera and clinical indices

**Figure 4:**
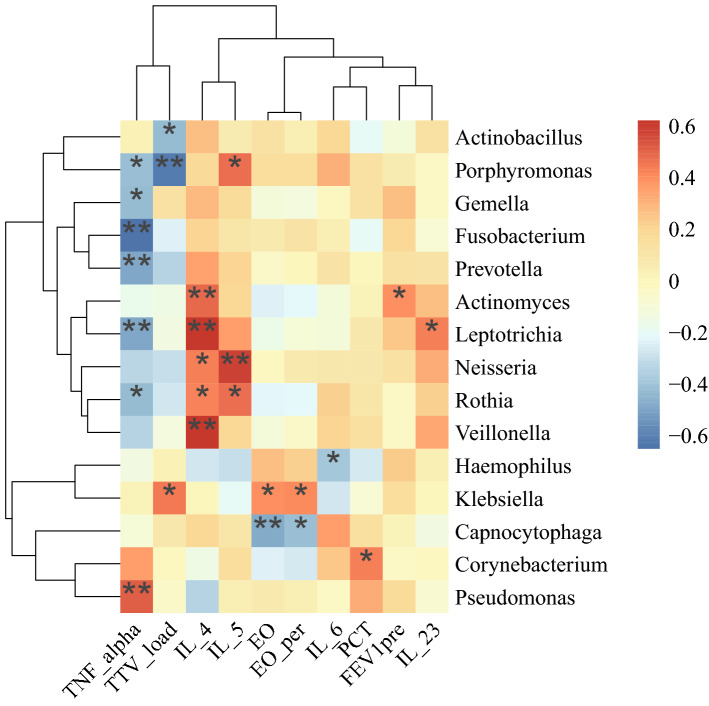
Heatmap of Spearman correlation analysis between the relative abundances of sputum microbiome and the clinical indices. *TNF* tumor necrosis factor, *IL* interleukin, *TTV* torque Teno virus, *PCT* procalcitonin, *EO* eosinophils. *p < 0.05, **p < 0.01.

The observed links between the respiratory microbial community and disease states prompted us to examine the interactions between taxa and clinical features during acute exacerbation. Previously, we found the Torque Teno Virus (TTV) DNA load is correlated with pulmonary function and COPD severity^18^; therefore. TTV was also included in the association analysis. The taxa analysed were the dominant genera during acute exacerbation and the top 15 genera in terms of abundance. The results showed significant positive correlations between the abundance of *Corynebacterium* and procalcitonin (PCT), abundance of *Pseudomonas* and TNF-α, abundance of *Klebsiella* and the percentage of eosinophils, and TTV DNA load. There were multiple moderate positive correlations between *Leptotrichia*, *Veillonella*, *Neisseria*, *Rothia*, and *Porphyromonas* and IL-23, IL-4, and IL-5 levels. However, the abundance of *Prevotella*, *Leptotrichia*, *Gemella*, *Rothia*, *Porphyromonas*, and *Fusobacterium* was significantly negatively correlated with TNF-α. No significant association was found between taxa and lung function (Table S7 and Fig. 4).

### Functional prediction of the COPD airway microbiome

The functional capacities of the bacterial communities in different disease states were analysed using PICRUSt2. Most functions decreased during acute exacerbation, except for ppGpp, fatty acid super-pathway, and mycolate biosynthesis. Degradation of l-arginine, tyrosine, l-ornithine, and androstenedione pathways were enhanced during acute exacerbation unlike during the stable period. Meanwhile, during the stable period, glucose oxidation, and CDP-archaea, l-tryptophan, and primordial inositol biosynthesis pathways were enhanced (Fig. S2). Furthermore, there was no significant difference in the relative abundance of the phenotypes among the three groups based on BugBase (Table S8).

### Unsupervised clustering revealed four distinct microbial clusters

**Figure 5:**
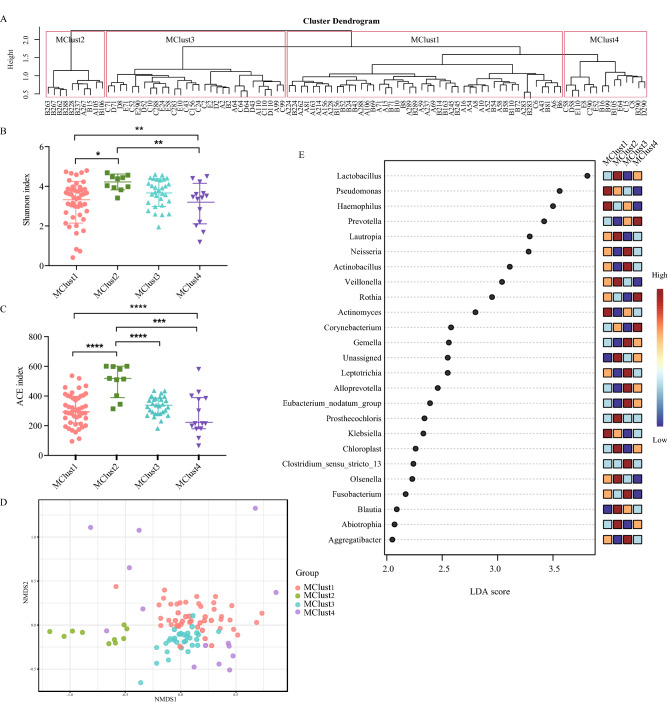
Characteristics of respiratory microbiome in four microbial clusters (MClusters). (**A**) Unsupervised clustering of respiratory microbiome based on Jaccard distance. (**B**,**C**) The Shannon index and ACE index among the four clusters. (**D**) The NMDS plot based on Bray Curtis distances. (**E**) Histogram of LDA values of LEfSe analysis of respiratory microbiome in clusters (*p* < 0.05 by the Wilcoxon test). *NMDS* non-metric multi-dimensional scaling, *LDA* linear discriminant analysis, *LEfSe* linear discriminant analysis effect size.

Based on the bacterial community information obtained by 16S rRNA gene sequencing, unsupervised cluster analysis was performed using the Jaccard similarity index, and four microbial clusters (MClusters)—MClus1, MClus2, MClus3, and MClus4—were obtained (Fig. 5A, Table S9). MClus1 and MClus2 were mainly composed of acute exacerbation and post-therapy communities, whereas MClus3 and MClus4 were mainly composed of stable communities. We counted smoking status in each cluster and the results were shown in Table S10. Further, we assessed smoking status between different clusters and found no statistically significant differences (p = 0.429).

Alpha diversity analysis showed that MClus2 had the highest diversity, and MClus4 had the lowest diversity (Fig. 5B,C and Table S11). The community structures of the four clusters had a partial degree of differentiation (Fig. 5D). We further analysed the dominant genera and differences in cytokine levels among the four clusters (Fig. 5E, Table S12). MClus1 was characterised by the enrichment of *Pseudomonas* and *Haemophilus* and showed a high level of TNF-α. MClus2, which is characterised by enriched *Lactobacillus*, *Lautropia*, and *Veillonella*, showed high levels of IL-4, IL-5, and IL-25, which may be related to steroid therapy. The samples from the stable stage were divided into two clusters with different taxological compositions. MClus3 was characterised by an increased abundance of *Neisseria*, *Actinobacillus*, *Gemella*, and *Leptotrichia* and showed high levels of IL-4, IL-17A, and IL-23. MClus4 had an increased abundance of *Prevotella*, *Rothia*, and *Corynebacterium* and showed a high level of IL-6. These findings suggest that the microbial clusters may indicate different inflammatory endotypes. Nevertheless, no differences in clinical manifestations were found between MClus3 and MClus4.

## Discussion

Recently, an emerging “keystone species” hypothesis was proposed, suggesting that even small changes in the abundance of a few bacterial species could have profound effects on the structure of the entire microbiome, thereby altering the disease status of the host^19^. Other studies have suggested that differences in the quorum sensing mechanism between organisms could affect the pathogenicity or antibiotic resistance of bacteria^20^. It is important to consider the overall environment of the microbial community to better understand the pathogenesis of COPD.

In this study, we explored the airway microbiome and inflammatory reactions in different disease states of COPD. The results showed that there were different bacterial taxa and functional capacities in the respiratory microecology of COPD among the three disease states. Potential pathogens were enriched during exacerbations, and some genera were found to be predictors of exacerbation. Significant correlations were also found between the airway microbiome and systemic or local inflammatory indicators in the sputum or specific disease phenotypes. Furthermore, cluster analysis identified four distinct microbial clusters, among which MClus1 and MClus2 were mainly composed of acute exacerbation and post-therapy communities, whereas MClus3 and MClus4 were mainly composed of stable communities. This is in contrast to previous studies which found that taxa were relatively stable across different disease states^21^. Subjects in different clusters were characterised by different genera and cytokines, which may contribute to the pathogenesis of COPD and serve as potentially useful starting points for targeted and effective therapeutics for COPD.

Our data showed that the acute exacerbation group had reduced microbial diversity, consistent with previous studies^21^. Previous studies have shown that patients with advanced disease have significantly lower bacterial diversity and higher mortality^22^. Thus, microbial community diversity may be critical for maintaining respiratory health, playing roles in colonisation resistance, epithelial integrity, and immune regulation^23,24^. Proteobacteria were predominant during acute exacerbations, whereas Bacteroidetes and Firmicutes were predominant during the stable period, with a more balanced and diversified microbial community. Proteobacteria is predominant in patients with low eosinophil counts, chronic bronchitis symptoms, and frequent exacerbations, whereas Firmicutes was predominant in patients with milder presentations^3^.

The microbial composition during acute exacerbations differed significantly from that during the stable period. The abundances of *Haemophilus*, *Moraxella*, *Klebsiella*, *Pseudomonas*, and *Corynebacterium* increased during exacerbations. Among them, *Pseudomonas* and *Corynebacterium* were the most strongly associated with immune-inflammatory markers, including PCT and TNF-α, suggesting that these two genera play important roles in host-microbiome associations. The bacterial genera enriched in the stable period mainly included *Neisseria*, *Clostridium*, *Bacteroides*, which is consistent with the typical lung microbiota composition of healthy adults^25^. Moreover, a considerable proportion of the taxa that existed during the stable period were not detected during acute exacerbations, indicating that during acute exacerbations, there was an increased abundance of potential pathogens, accompanied by depletion of some colonising bacteria.

Increasing evidence suggests that the airway microbiome is associated with an inflammatory phenotype in COPD. Four microbial clusters were obtained by unsupervised cluster analysis, revealing distinct inflammation types. These findings can contribute to better understanding the pathophysiological basis of COPD and argue for the need to tailor therapies. MClust1, which was dominated by samples from the acute exacerbation period, had low species diversity, with *Pseudomonas*, *Haemophilus*, *Actinomyces*, and *Klebsiella* as the main genera, and the highest level of TNF-α, showing a Th1 inflammatory signature, consistent with a previous study^10^. *Haemophilus* and *Pseudomonas* are neutrophil-specific respiratory pathogens that induce proinflammatory host response^26^. In addition, multiple studies have shown that acute exacerbation has two distinct endotypes: bacteria-associated and eosinophil-associated exacerbations. The bacteria-associated exacerbation had decreased microbial diversity and increased Proteobacteria abundance^4,27^, and antibiotics prevented such exacerbations^28^. Our results were consistent with bacterial-related exacerbations, although no evidence of eosinophilic exacerbation was found.

Samples in the stable period were divided into two clusters. MClust3 was dominated by *Neisseria*, *Actinobacillus*, *Gemella*, and *Leptotrichia*, with significantly higher levels of IL-5, IL-4, IL-17A, and IL-23, suggesting Th2-related inflammation. *Gemella* was enriched in the eosinophil group^10^, and Th2 inflammation was predominantly associated with eosinophils. Treating eosinophilic inflammation in COPD patients with glucocorticoids can reduce the frequency of exacerbations^29^. Although no existing monoclonal antibody therapy targets the IL-5 cytokine and its receptor in COPD, decreasing IL-5 may be effective in reducing the risk of exacerbation in patients with eosinophilic asthma^30^. MClust4 was dominated by *Prevotella*, *Rothia*, and *Corynebacterium*, with elevated IL-6 levels and Th17-related inflammatory characteristics. *Prevotella* is a core genus of anaerobic bacteria in the oral microbiome and is a member of the adult lung microbiome^25^. It is enriched in patients with both common pneumonia and COVID-19 and is elevated in patients with persistent symptoms^31^. In mouse models, intermittent inhalation of the oral commensal *Prevotella melaninogenica* can lead to dysbiosis and low-level inflammation of the lower respiratory tract, reducing susceptibility to *Streptococcus pneumoniae* by activating the lung Th17 cell response^32^. *Prevotella* also induces COPD-like symptoms by activating neutrophils and increasing Toll-like receptor (TLR)-2 dependent cytokine expression^33^. These results suggest that commensal *Prevotella* not only aggravates airway immune responses but may also modulate pathogen-induced inflammatory responses. Thus, COPD may have distinct inflammatory endotypes, and anti-inflammatory therapy alone may be ineffective against chronic airway inflammation in COPD unless the underlying bacterial infections driving abnormal immune responses are controlled. Targeted therapies can improve chronic inflammation and the long-term prognosis of patients. However, in recent studies, the effects of anti-inflammatory or antibiotic treatments are uncertain. For example, macrolide treatment reduces the relative abundance of *Haemophilus* while simultaneously increasing the abundance of other *Proteobacteria*, including *Pseudomonas*^34^. Therefore, exploring the effects of treatments combined with microbial information is vital.

This study had some limitations. First, we mainly focused on patients with COPD without healthy controls. Second, owing to the relatively low depth of 16S rRNA sequencing, information at the species level is lacking. In addition, 16S rRNA sequencing mainly focused on bacteria, so it was impossible to explore the characteristics of viral and fungal composition and their specific relationship with diseases. Due to the lack of sufficient numbers of patients who could provide longitudinal specimens, we did not perform a within subject analysis. And, since the samples in the acute exacerbation state and the stable state were not one-to-one corresponding, we cannot analyze the respiratory microbiome in the exacerbation belonging to the two inflammatory endotypes. However, our study illustrated that there may be different inflammation types during the stable state, and that these inflammation types may vary in terms of acute exacerbation, clinical features, and prognosis of COPD. Finally, despite efforts made in quality control, oral contamination cannot be completely excluded. Overall, this study suggests that respiratory tract microecology is associated with host immunity and disease status. We look forward to future multicentre cohort studies with larger populations to further validate our findings. Simultaneously, multiple omics, such as metabolomics, metagenomics, or metatranscriptomics, can be combined to further explore the role of respiratory microecology in the pathogenesis of COPD.

## Conclusions

Our results showed that the composition of microbiome altered in different disease states of COPD and the AECOPD is characterised by reduced microbial diversity and increased abundances of potentially pathogenic bacteria, which were strongly associated with immune-inflammatory markers. Inflammatory endotypes with different microbial compositions and inflammatory features were found in the stable period, thus arguing for the tailor therapy on the specific endotype and related inflammatory status exhibited by the subjects.

## Supplementary Information

Supplementary Information.

## Data Availability

The datasets presented in this study can be found in online repositories. The names of the repositories/repositories and accession number(s) can be found below: https://www.ncbi.nlm.nih.gov/, PRJNA861712. The data supporting the findings of this study are available by contacting the corresponding author.

## Abbreviations

AE: Acute exacerbation
ANOVA: One-way analysis of variance
AUC: Area under the ROC curve
ASV: Amplicon sequence variables
BMI: Body mass index
CAT: COPD assessment test
COPD: Chronic obstructive pulmonary disease
COVID-19: Coronavirus disease 2019
CRP: C-reactive protein
EO: Eosinophils
FDR: False discovery rates
FeNO: Fractional exhaled nitric oxide
FEV1: Forced expiratory volume in 1 s
FVC: Forced vital capacity
GOLD: Global initiative for chronic obstructive lung disease
IFN: Interferon
IL: Interleukin
IQR: Interquartile range
LEfSe: Linear discriminant analysis effect size
LDA: Linear discriminant analysis
mMRC: Modified British medical research council
NMDS: Non-metric multi-dimensional Scaling
OTU: Operational taxonomic unit
PCoA: Principal coordinate analysis
PCT: Procalcitonin
ROC: Receiver operating characteristic
RV: Residual volume
TLC: Total lung capacity
TLR2: Toll-like receptor 2
TNF: Tumor necrosis factor
TTV: Torque teno virus
WBC: White blood cell count
6MWD: 6-Minutes walk test distance
