# Supplementary material for: Lung microbiome and cytokine profiles in different disease states of COPD: a cohort study
Source: Sci Rep. 2023 Apr 7;13:5715. doi: 10.1038/s41598-023-32901-0 (PMC10080507; doi:10.1038/s41598-023-32901-0)
Supplement: Supplementary file 1 — Supplementary Information. [file 41598_2023_32901_MOESM1_ESM.docx]

**Supplementary materials**

**Contents of Supplements**

[Supplement 1 Supplementary Methods 2](#_Toc109329656)

[Supplement 2 Supplementary Figures 6](#_Toc109329657)

[Supplement 3 Supplementary Tables 9](#_Toc109329658)

# Supplement 1 Supplementary Methods

**DNA extraction, Amplicon Generation and Library preparation**

Detailed procedure for sputum specimens processing has been described in a published study^1^. After centrifuged the supernatants and precipitates were preserved separately at -80°C.The whole genome DNA was extracted by CTAB method in precipitates. Specific primers (341F and 806R) with Barcode were used to amplify 16S rRNA genes of V3-V4 region. All PCR reactions were carried out in 30μL reactions with 15μL of Phusion® High-Fidelity PCR Master Mix (New England Biolabs); 0.2μM of forward and reverse primers, and about 10 ng template DNA. Thermal cycling consisted of initial denaturation at 98 ℃ for 1 min, followed by 30 cycles of denaturation at 98 ℃ for 10 s, annealing at 50 ℃ for 30 s, and elongation at 72 ℃ for 30 s. Finally, 72 ℃ for 5 min. Mix same volume of 1X loading buffer (contained SYB green) with PCR products and operate electrophoresis on 2% agarose gel for detection. Samples with bright main strip between 400-450bp were chosen for further experiments. Following manufacturer’s recommendations, sequencing libraries were generated with NEBNext Ultra^TM^ IIDNA Library Prep Kit (Cat No. E7645). The library quality was evaluated on the Qubit 2.0 Fluorometer (Thermo Scientific) and Agilent Bioanalyzer 2100 system.

**Sequencing analysis**

In order to analyze the diversity, richness and uniformity of the communities in the samples, alpha diversity was calculated from 3 indices in QIIME2, including Observed_otus, Shannon, and Paith_PD. Beta diversity was calculated to evaluate the complexity of the community composition and compare the differences between samples. Principal Coordinate Analysis (PCoA) based on beta diversity was performed. Adonis was used to analyze the significance of community structure differences between groups. Heatmap was generated through ppheatmap R package. Differential bacterial taxa among groups were assessed using linear discriminant analysis (LDA) effect size (LEfSe) and Limma R package. In the LEfSe analysis, genera with LDA >2 and *p* < 0.05 were differential. Further, PICRUSt2 software (Version 2.1.2-b) was used for functional prediction. The BugBase phenotypic database (https://bugbase.cs.umn.edu/) was used to predict bacterial phenotypes. Unsupervised hierarchical clustering with Ward’s minimum variance was performed with the hclust function of the R Cluster package. The Nbclust package was used to evaluate the optimal clusters, and the Jaccard similarity index was used to assess the cluster stability.

**Analysis of contaminations**

Before sequencing, we first extracted genomic DNA from sputums and performed PCR Products quantification and qualification. Genomic DNA was diluted with at least 3 biological replicates. Sterilized ddH_2_O was used as the negative reagent as the control. Meanwhile, primer dilution was used as sterilized ddH_2_O. Based on the PCR amplified products used in the study, 2% concentration of agarose gel was selected for electrophoretic detection of all products, and follow-up experiments were conducted when there was no strip in the negative control. In order to control the influence of sample contamination, aseptic operations were carried out throughout the sample collection and experiment, and negative controls were set at each step of the experiment. At the same time, further analysis was performed to ensure that the potential risks of contamination were minimized. The results of this study were compared with the 92 contamination genera detected in the negative sequencing blank control by Salter et al^2^. We failed to detect 70 out of the contaminant genera in our dataset (Table S2). Among the remaining genera found in our data, except for some known pulmonary bacteria or pathogens (including *Corynebacterium*, *Streptococcus*, *Pseudomonas*, *Abiotrophia* and *Aeromonas*), no genus had an average relative abundance greater than 0.0009, or had a relative abundance greater than 0.1 in a particular sample.

**Cytokine measurements**

A total of 12 cytokines were measured in the sputum supernatants using Luminex Human Magnetic Assay Kit (LXSAHM-12; R&D Systems, Minneapolis, MN, USA), including Th1 (TNF-α, IFN-γ, and IL-2), Th2 (IL-4, IL-5, and IL-25), Th17 (IL-6, IL-17A, IL-21, IL-22, and IL-23), and Treg (IL-10) cytokines, according to the manufacturer’s instructions.

**TTV Quantification**

The method of TTV quantification was described in our previous study^3^. TaqMan real-time PCR amplification and the standard curve method were used to measure the TTV loads. The quantitative assays were performed under the following conditions: 95.0°C for 30 s, followed by 40 amplification cycles of 5 s at 95.0°Cand 30 s at 60.0°C. We designed primers and probes according to the relatively conserved PCR sequence (EcoRI to HindIII, 251 bp) of the TTV genome (pcDNA™ 3.1/myc-His(-) A plasmid) and used them to generate the standard curve. The Ct values of the standard curve were obtained using seven 10-fold dilutions. The Ct values generated by the 10-fold dilution sequence were regressed, and the correlation coefficient was 0.99. Specimens of patients were tested at least in duplicate.

**Statistical Analysis**

IBM SPSS Statistics 25.0 and GraphPad Prism Version 7.0, were used for statistical analysis. The Shapiro-Wilk test is used to assess the normality of clinical and experimental data. Continuous variables are expressed as mean ± standard deviation for normal distribution and as median and quartile spacing (IQR, 25th and 75th percentiles) for non-normal distribution. Categorical variables are expressed in numbers (percentages). T test or one-way analysis of variance (ANOVA) was used for normally distributed continuous variables, and Mann-Whitney U test or Kruskal-Wallis test was used for non-normally distributed continuous variables. Fisher's exact test, Chi-square test, or trend Chi-square test were used to analyze categorical variables. Spearman rank test was used to analyze the correlation between variables. ROC analysis was used to screen for genera that predicted acute exacerbations. False discovery rate (FDR) was used to correct *p* values, and *p* < 0.05 was considered statistically significant.

# Supplement 2 Suppl
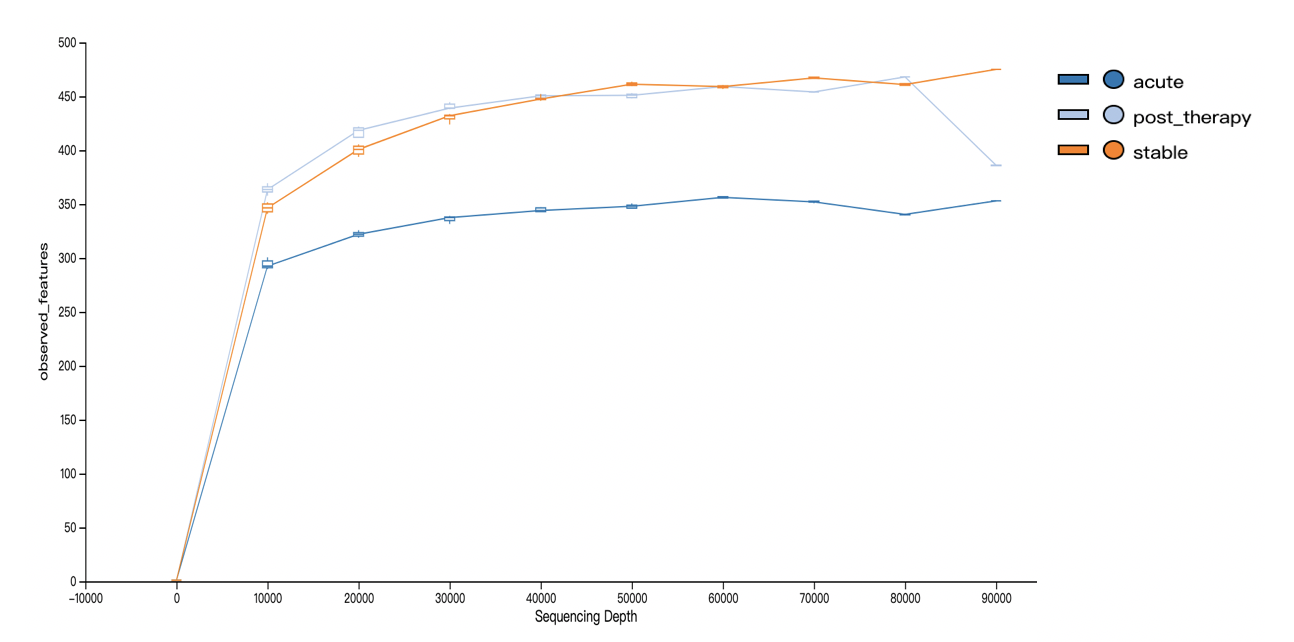
ementary Figures

**Figure S1.** Species sparsity curve. The abscissa is the amount of sequencing data, and the ordinate is the number of species observed. With the increase of sequencing depth, the curve tends to be flat, indicating that the amount of sequencing data is sufficient for research and analysis.


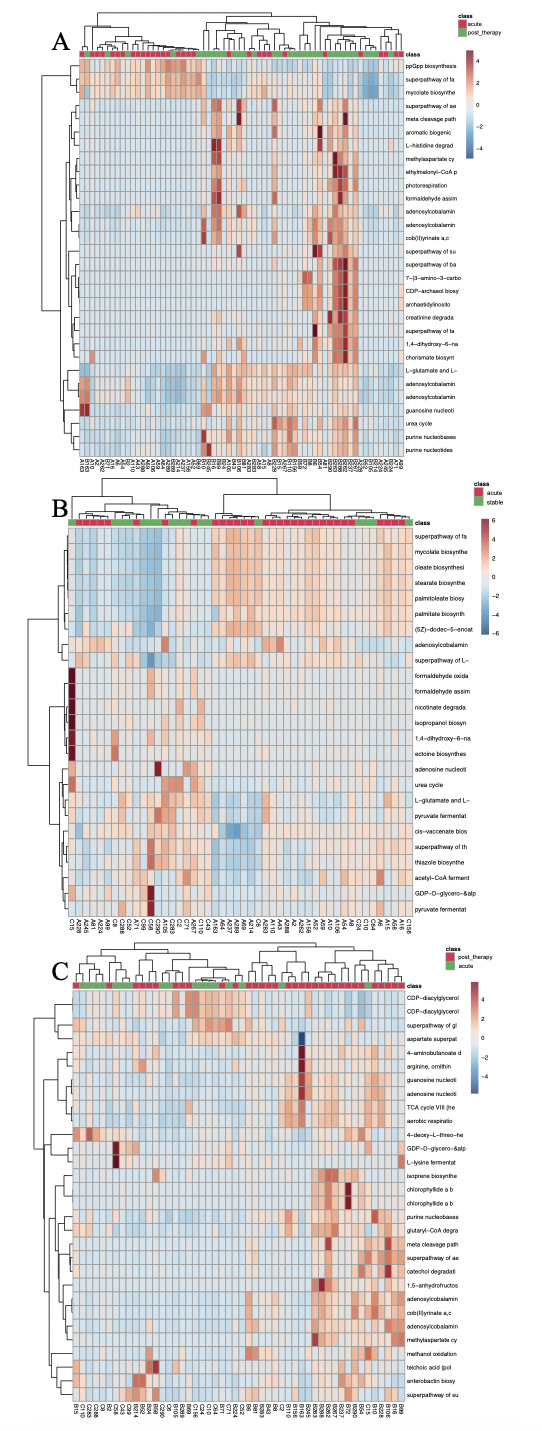


**Figure S2.** Function prediction of PICRUSt2 genes in different disease status. The red and blue squares indicate functionality rising or falling in the group, respectively. Comparison of function between acute exacerbation and post therapy (A), acute exacerbation and stable phase (B), post therapy and stable phase (C).

# Supplement 3 Supplementary Tables

**Table S1.** The Adonis test based on four distance indices

| Indices | p values | post hoc test significant values | |
| --- | --- | --- | --- |
| Bray-Curtis | 0.001 | Acute vs Post-therapy | 0.009 |
|  |  | Acute vs Stable | 0.008 |
|  |  | Post-therapy vs Stable | 0.001 |
| Jaccard | 0.001 | Acute vs Post-therapy | 0.001 |
|  |  | Acute vs Stable | 0.001 |
|  |  | Post-therapy vs Stable | 0.001 |
| Weighted-Unifrac | 0.040 | Acute vs Post-therapy | 0.117 |
|  |  | Acute vs Stable | 0.161 |
|  |  | Post-therapy vs Stable | 0.059 |
| Unweighted-Unifrac | 0.001 | Acute vs Post-therapy | 0.003 |
|  |  | Acute vs Stable | 0.001 |
|  |  | Post-therapy vs Stable | 0.001 |

**Table S2.** The occurrence and average relative abundance of contamination genera detected in the negative sequencing blank controls compared to Salter et al.

| **Genus** | **Occurrence (rel abundance > 0)** | **Occurrence (rel abundance > 0.1)** | **Average relative abundance** |
| --- | --- | --- | --- |
| **Alphaproteobacteria** |  |  |  |
| *Afipia* | 0 | 0 | 0 |
| *Aquabacterium* | 0.06 | 0 | 6.84351E-05 |
| *Asticcacaulis* | 0 | 0 | 0 |
| *Aurantimonas* | 0 | 0 | 0 |
| *Beijerinckia* | 0 | 0 | 0 |
| *Bosea* | 0 | 0 | 0 |
| *Bradyhizobium* | 0 | 0 | 0 |
| *Brevundimonas* | 0.47 | 0 | 0.000482842 |
| *Caulobacter* | 0 | 0 | 0 |
| *Craurococcus* | 0 | 0 | 0 |
| *Devosia* | 0 | 0 | 0 |
| *Hoeflea* | 0 | 0 | 0 |
| *Mesorhizobium* | 0 | 0 | 0 |
| *Methylobacterium* | 0 | 0 | 0 |
| *Novosphingobioum* | 0 | 0 | 0 |
| *Ochrobactrum* | 0 | 0 | 0 |
| *Paracoccus* | 0 | 0 | 0 |
| *Pedomicrobiom* | 0 | 0 | 0 |
| *Phyllobacterium* | 0 | 0 | 0 |
| *Rhizobium* | 0 | 0 | 0 |
| *Roseomonas* | 0 | 0 | 0 |
| *Sphingobium* | 0 | 0 | 0 |
| *Sphingomonas* | 0.11 | 0 | 0.000150887 |
| *Sphingopyxis* | 0.03 | 0 | 1.69022E-05 |
| ***Betaproteobacteria*** |  |  |  |
| *Acidovorax* | 0.08 | 0 | 0.000105041 |
| *Azoarcus* | 0 | 0 | 0 |
| *Azospira* | 0 | 0 | 0 |
| *Burkholderia* | 0 | 0 | 0 |
| *Comamonas* | 0.58 | 0 | 0.000377037 |
| *Cupriavidus* | 0.03 | 0 | 3.10823E-05 |
| *Curvibacter* | 0 | 0 | 0 |
| *Delftia* | 0.30 | 0 | 0.000228837 |
| *Duganella* | 0 | 0 | 0 |
| *Herbaspirillum* | 0 | 0 | 0 |
| *Janthinobacterium* | 0 | 0 | 0 |
| *Kingella* | 0.44 | 0 | 0.000655769 |
| *Leptothrix* | 0 | 0 | 0 |
| *Limnobacter* | 0 | 0 | 0 |
| *Massilia* | 0 | 0 | 0 |
| *Methylophilus* | 0 | 0 | 0 |
| *Methyloversatilis* | 0 | 0 | 0 |
| *Oxalobacter* | 0 | 0 | 0 |
| *Pelomonas* | 0 | 0 | 0 |
| *Polaromonas* | 0 | 0 | 0 |
| *Ralstonia* | 0.63 | 0 | 0.00070424 |
| *Schlegelella* | 0.13 | 0 | 5.04861E-05 |
| *Sulfuritalea* | 0 | 0 | 0 |
| *Undibacterium* | 0 | 0 | 0 |
| *Variovorax* | 0 | 0 | 0 |
| **Gammaproteobacteria** |  |  |  |
| *Acinetobacter* | 0.20 | 0 | 0.00011567 |
| *Enhydrobacter* | 0.32 | 0 | 0.000107154 |
| *Enterobacter* | 0.08 | 0 | 7.01076E-05 |
| *Escherichia* | 0 | 0 | 0 |
| *Nevskia* | 0 | 0 | 0 |
| *Pseudomonas* | 0.56 | 0.03 | 0.023153633 |
| *Pseudoxanthomonas* | 0 | 0 | 0 |
| *Psychobacter* | 0 | 0 | 0 |
| *Stenotrophomonas* | 0 | 0 | 0 |
| *Xanthomonas* | 0 | 0 | 0 |
| **Actinobacteria** |  |  |  |
| *Aeromonas* | 0.25 | 0 | 0.002173028 |
| *Arthrobacter* | 0 | 0 | 0 |
| *Beutenbergia* | 0 | 0 | 0 |
| *Brevibacterium* | 0 | 0 | 0 |
| *Corynebacterium* | 0.98 | 0.01 | 0.010510226 |
| *Curtobacterium* | 0 | 0 | 0 |
| *Dietzia* | 0 | 0 | 0 |
| *Geodermatophilus* | 0 | 0 | 0 |
| *Janibacter* | 0 | 0 | 0 |
| *Kocuria* | 0 | 0 | 0 |
| *Microbacterium* | 0 | 0 | 0 |
| *Micrococcus* | 0 | 0 | 0 |
| *Microlunatus* | 0 | 0 | 0 |
| *Patulibacter* | 0 | 0 | 0 |
| *Propionibacterum* | 0 | 0 | 0 |
| *Rhodococcus* | 0 | 0 | 0 |
| *Tsukamurella* | 0 | 0 | 0 |
| **Firmicutes** |  |  |  |
| *Abiotrophia* | 0.86 | 0 | 0.002149565 |
| *Bacillus* | 0.20 | 0 | 0.000106134 |
| *Brevibacillus* | 0 | 0 | 0 |
| *Brochothrix* | 0.17 | 0 | 0.000856828 |
| *Facklamia* | 0 | 0 | 0 |
| *Paenibacillus* | 0.08 | 0 | 4.62602E-05 |
| *Streptococcus* | 1.00 | 0.66 | 0.229807475 |
| **Bacteroidetes** |  |  |  |
| *Chryseobacterium* | 0 | 0 | 0 |
| *Dyadobacter* | 0 | 0 | 0 |
| *Flavobacterium* | 0 | 0 | 0 |
| *Hydrotalea* | 0 | 0 | 0 |
| *Niatella* | 0 | 0 | 0 |
| *Olivibacter* | 0 | 0 | 0 |
| *Pedobacter* | 0 | 0 | 0 |
| *Wautersiella* | 0 | 0 | 0 |
| **Deinococcus-Thermus** |  |  |  |
| *Deinococcus* | 0 | 0 | 0 |

**Table S3.** Differential taxa of acute exacerbation and stable phase obtained by Limma analysis

| **Genus** | **logFC** | ***p*-value** | **FDR** |
| --- | --- | --- | --- |
| *Haemophilus* | 1.576 | 0.00912361 | 0.04105624 |
| *Pseudomonas* | 7.643 | 3.63E-06 | 6.53E-05 |
| *Corynebacterium* | 3.487 | 3.00E-08 | 1.75E-06 |
| *Chloroplast* | 5.108 | 1.36E-05 | 0.00015886 |
| *Klebsiella* | 5.746 | 0.00042184 | 0.00266786 |
| *Capnocytophaga* | 1.797 | 0.00017349 | 0.00144984 |
| *Clostridium_sensu_stricto_13* | -12.775 | 2.07E-07 | 8.09E-06 |
| *Eubacterium_nodatum_group* | -2.001 | 9.40E-05 | 0.00087948 |
| *Faecalibacterium* | -3.838 | 1.89E-05 | 0.00020148 |
| *Moraxella* | 5.064 | 0.00028666 | 0.00209619 |
| *Bacteroides* | -4.02 | 1.26E-08 | 9.81E-07 |
| *Bordetella* | -10.431 | 4.12E-05 | 0.00040208 |
| *Olsenella* | 5.115 | 0.01458981 | 0.05786466 |
| *hgcI_clade* | -6.027 | 0.02234425 | 0.08043931 |
| *Staphylococcus* | -3.309 | 0.01039255 | 0.04503436 |
| *Roseburia* | -4.802 | 0.00021656 | 0.00168917 |
| *Kingella* | -4.825 | 3.49E-06 | 6.53E-05 |
| *F0332* | 6.916 | 1.49E-05 | 0.00016579 |
| *Butyrivibrio* | -1.725 | 0.0030952 | 0.01478116 |
| *Vibrio* | 5.114 | 4.88E-07 | 1.37E-05 |
| *Lactococcus* | -4.564 | 0.01831413 | 0.06912106 |
| *Lachnospiraceae_UCG-003* | -8.913 | 1.34E-05 | 0.00015886 |
| *Ruminococcus* | -4.089 | 0.02642369 | 0.09092859 |
| *Clostridia_vadinBB60_group* | 2.997 | 0.0006436 | 0.00386161 |
| *Ruminococcus_gnavus_group* | -4.107 | 9.44E-06 | 0.00012991 |
| *Eubacterium_eligens_group* | -4.231 | 0.00625316 | 0.0292648 |
| *Lachnoclostridium* | -5.5 | 6.14E-08 | 2.87E-06 |
| *Ileibacterium* | 5.219 | 7.14E-06 | 0.00011136 |
| *Lachnospira* | -6.285 | 5.28E-07 | 1.37E-05 |
| *Lachnospiraceae_NK4A136_group* | -8.113 | 8.60E-06 | 0.00012583 |
| *Butyricicoccus* | -8.622 | 2.15E-12 | 5.04E-10 |
| *Dolosigranulum* | 2.391 | 0.02276156 | 0.08070006 |
| *Enterococcus* | -4.533 | 0.01540251 | 0.05908504 |
| *Phocaeicola* | 2.678 | 0.03002425 | 0.09895315 |
| *Clostridium_sensu_stricto_1* | -2.589 | 0.01110388 | 0.04558433 |
| *Clostridium_sensu_stricto_18* | -7.774 | 0.00020517 | 0.00165547 |
| *Flavonifractor* | -7.161 | 2.50E-11 | 2.93E-09 |
| *Sporolactobacillaceae* | 5.745 | 1.22E-05 | 0.00015864 |
| *Clostridium_sensu_stricto_7* | -7.503 | 0.00024374 | 0.00183983 |
| *Ruminiclostridium* | -7.212 | 0.00030032 | 0.00212391 |
| *Methanobrevibacter* | -6.948 | 0.00110264 | 0.00600042 |
| *Prevotellaceae_NK3B31_group* | -7.568 | 0.0007565 | 0.00437294 |
| *Parabacteroides* | -6.499 | 4.23E-06 | 7.08E-05 |
| *Bacillus* | 4.696 | 7.13E-07 | 1.67E-05 |
| *Paenibacillus* | 4.558 | 0.00081191 | 0.00452348 |
| *Aminicenantales* | -7.392 | 0.0019644 | 0.00999283 |
| *Clostridium_innocuum_group* | -5.853 | 4.24E-07 | 1.37E-05 |
| *Slackia* | 3.634 | 0.02382637 | 0.08321449 |
| *Alistipes* | -3.716 | 0.01905276 | 0.07076738 |
| *Dysgonomonas* | -5.051 | 0.01098572 | 0.04558433 |
| *Lachnospiraceae_ND3007_group* | -5.627 | 0.0001242 | 0.00107637 |
| *Lachnospiraceae_UCG-004* | -6.064 | 0.00038403 | 0.00249618 |
| *NK4A214_group* | -6.951 | 0.00035225 | 0.00235501 |
| *UCG-005* | -7.314 | 0.0007662 | 0.00437294 |
| *CAG-352* | -4.062 | 0.01407823 | 0.05679837 |
| *Caproiciproducens* | -7.151 | 0.0003086 | 0.00212391 |
| *Acidaminococcus* | -5.093 | 0.01086158 | 0.04558433 |
| *Methanosaeta* | -7.628 | 0.00166901 | 0.00887611 |
| *Mitochondria* | 5.989 | 0.00050475 | 0.00310819 |
| *Novosphingobium* | -8.368 | 0.01512072 | 0.05897082 |
| *Acidovorax* | -6.962 | 0.00273431 | 0.01332974 |
| *Comamonas* | 2.924 | 0.00010084 | 0.00090753 |
| *Limnohabitans* | -9.272 | 0.01989246 | 0.07273181 |
| *Sutterella* | -7.681 | 1.15E-06 | 2.45E-05 |
| *Cardiobacterium* | 1.691 | 0.00185701 | 0.00965646 |
| *Mangrovibacter* | -9.786 | 3.59E-05 | 0.00036484 |
| *Proteus* | -9.337 | 0.00250147 | 0.01245415 |
| *Psychrobacter* | -4.564 | 0.02888488 | 0.09690885 |
| *Ahniella* | 2.807 | 0.01001147 | 0.0442016 |
| *Sphaerochaeta* | 4.325 | 0.02898983 | 0.09690885 |
| *Akkermansia* | 5.114 | 0.00652772 | 0.02995071 |

**Table S4.** Differential taxa of acute exacerbation and post therapy obtained by Limma analysis

| \| **Genus** \| **logFC** \| ***p*-value** \| **FDR** \| \| --- \| --- \| --- \| --- \| \| *CL500-29_marine_group* \| -6.419 \| 0.02060238 \| 0.07089644 \| \| *Actinomyces* \| -1.461 \| 0.00192012 \| 0.00976758 \| \| *F0332* \| 4.099 \| 0.00168604 \| 0.00896665 \| \| *Mobiluncus* \| -4.147 \| 0.0322186 \| 0.0901895 \| \| *Corynebacterium* \| 2.23 \| 0.00045901 \| 0.00394385 \| \| *Nakamurella* \| -5.356 \| 0.00027551 \| 0.00306993 \| \| *Kineosphaera* \| -5.638 \| 0.00175975 \| 0.00915069 \| \| *Aurantimicrobium* \| -6.87 \| 0.01308136 \| 0.04709289 \| \| *Cutibacterium* \| -1.34 \| 0.02723192 \| 0.08025606 \| \| *Micropruina* \| -8.044 \| 0.00036657 \| 0.00357411 \| \| *Olsenella* \| -3.293 \| 0.02631932 \| 0.08019075 \| \| *Collinsella* \| -3.09 \| 0.0399412 \| 0.09942809 \| \| *Parabacteroides* \| -3.464 \| 0.00164597 \| 0.00896665 \| \| *Bergeyella* \| 3.59 \| 0.0004962 \| 0.00400381 \| \| *Lentimicrobiaceae* \| -4.787 \| 0.000926 \| 0.005556 \| \| *Prosthecochloris* \| -10.341 \| 1.85E-05 \| 0.0008667 \| \| *Caldithrix* \| -7.431 \| 0.00016252 \| 0.00253528 \| \| *Calditrichaceae* \| -6.532 \| 0.00038775 \| 0.00362936 \| \| *Arcobacter* \| -4.92 \| 0.0329004 \| 0.0901895 \| \| *Helicobacter* \| -3.396 \| 0.01797897 \| 0.06374362 \| \| *Sulfurovum* \| -9.161 \| 1.43E-05 \| 0.00083858 \| \| *JG30-KF-CM45* \| -5.656 \| 0.02673025 \| 0.08019075 \| \| *Desulfatiglans* \| -5.682 \| 0.00061215 \| 0.0043407 \| \| *Clostridia_vadinBB60_group* \| 2.997 \| 0.0006436 \| 0.00386161 \| \| *Ruminococcus_gnavus_group* \| -4.107 \| 9.44E-06 \| 0.00012991 \| \| *Eubacterium_eligens_group* \| -4.231 \| 0.00625316 \| 0.0292648 \| \| *Lachnoclostridium* \| -5.5 \| 6.14E-08 \| 2.87E-06 \| \| *Ileibacterium* \| 5.219 \| 7.14E-06 \| 0.00011136 \| \| *Lachnospira* \| -6.285 \| 5.28E-07 \| 1.37E-05 \| \| *Lachnospiraceae_NK4A136_group* \| -8.113 \| 8.60E-06 \| 0.00012583 \| \| *Butyricicoccus* \| -8.622 \| 2.15E-12 \| 5.04E-10 \| \| *Dolosigranulum* \| 2.391 \| 0.02276156 \| 0.08070006 \| \| *Enterococcus* \| -4.533 \| 0.01540251 \| 0.05908504 \| \| *Phocaeicola* \| 2.678 \| 0.03002425 \| 0.09895315 \| \| *Clostridium_sensu_stricto_1* \| -2.589 \| 0.01110388 \| 0.04558433 \| \| *Clostridium_sensu_stricto_18* \| -7.774 \| 0.00020517 \| 0.00165547 \| \| *Flavonifractor* \| -7.161 \| 2.50E-11 \| 2.93E-09 \| \| *Sporolactobacillaceae* \| 5.745 \| 1.22E-05 \| 0.00015864 \| \| *Clostridium_sensu_stricto_7* \| -7.503 \| 0.00024374 \| 0.00183983 \| \| *Ruminiclostridium* \| -7.212 \| 0.00030032 \| 0.00212391 \| \| *Methanobrevibacter* \| -6.948 \| 0.00110264 \| 0.00600042 \| \| *Prevotellaceae_NK3B31_group* \| -7.568 \| 0.0007565 \| 0.00437294 \| \| *Parabacteroides* \| -6.499 \| 4.23E-06 \| 7.08E-05 \| \| *Bacillus* \| 4.696 \| 7.13E-07 \| 1.67E-05 \| \| *Paenibacillus* \| 4.558 \| 0.00081191 \| 0.00452348 \| \| *Aminicenantales* \| -7.392 \| 0.0019644 \| 0.00999283 \| \| *Clostridium_innocuum_group* \| -5.853 \| 4.24E-07 \| 1.37E-05 \| \| *Slackia* \| 3.634 \| 0.02382637 \| 0.08321449 \| \| *Alistipes* \| -3.716 \| 0.01905276 \| 0.07076738 \| \| *Dysgonomonas* \| -5.051 \| 0.01098572 \| 0.04558433 \| \| *Lachnospiraceae_ND3007_group* \| -5.627 \| 0.0001242 \| 0.00107637 \| \| *Lachnospiraceae_UCG-004* \| -6.064 \| 0.00038403 \| 0.00249618 \| \| *NK4A214_group* \| -6.951 \| 0.00035225 \| 0.00235501 \| \| *UCG-005* \| -7.314 \| 0.0007662 \| 0.00437294 \| \| *CAG-352* \| -4.062 \| 0.01407823 \| 0.05679837 \| \| *Caproiciproducens* \| -7.151 \| 0.0003086 \| 0.00212391 \| \| *Acidaminococcus* \| -5.093 \| 0.01086158 \| 0.04558433 \| \| *Methanosaeta* \| -7.628 \| 0.00166901 \| 0.00887611 \| \| *Mitochondria* \| 5.989 \| 0.00050475 \| 0.00310819 \| \| *Novosphingobium* \| -8.368 \| 0.01512072 \| 0.05897082 \| \| *Acidovorax* \| -6.962 \| 0.00273431 \| 0.01332974 \| \| *Comamonas* \| 2.924 \| 0.00010084 \| 0.00090753 \| \| *Limnohabitans* \| -9.272 \| 0.01989246 \| 0.07273181 \| \| *Sutterella* \| -7.681 \| 1.15E-06 \| 2.45E-05 \| \| *Cardiobacterium* \| 1.691 \| 0.00185701 \| 0.00965646 \| \| *Mangrovibacter* \| -9.786 \| 3.59E-05 \| 0.00036484 \| \| *Proteus* \| -9.337 \| 0.00250147 \| 0.01245415 \| \| *Psychrobacter* \| -4.564 \| 0.02888488 \| 0.09690885 \| \| *Ahniella* \| 2.807 \| 0.01001147 \| 0.0442016 \| \| *Sphaerochaeta* \| 4.325 \| 0.02898983 \| 0.09690885 \| \| *Akkermansia* \| 5.114 \| 0.00652772 \| 0.02995071 \| |
| --- | --- | --- | --- | --- | --- | --- | --- | --- | --- | --- | --- | --- | --- | --- | --- | --- | --- | --- | --- | --- | --- | --- | --- | --- | --- | --- | --- | --- | --- | --- | --- | --- | --- | --- | --- | --- | --- | --- | --- | --- | --- | --- | --- | --- | --- | --- | --- | --- | --- | --- | --- | --- | --- | --- | --- | --- | --- | --- | --- | --- | --- | --- | --- | --- | --- | --- | --- | --- | --- | --- | --- | --- | --- | --- | --- | --- | --- | --- | --- | --- | --- | --- | --- | --- | --- | --- | --- | --- | --- | --- | --- | --- | --- | --- | --- | --- | --- | --- | --- | --- | --- | --- | --- | --- | --- | --- | --- | --- | --- | --- | --- | --- | --- | --- | --- | --- | --- | --- | --- | --- | --- | --- | --- | --- | --- | --- | --- | --- | --- | --- | --- | --- | --- | --- | --- | --- | --- | --- | --- | --- | --- | --- | --- | --- | --- | --- | --- | --- | --- | --- | --- | --- | --- | --- | --- | --- | --- | --- | --- | --- | --- | --- | --- | --- | --- | --- | --- | --- | --- | --- | --- | --- | --- | --- | --- | --- | --- | --- | --- | --- | --- | --- | --- | --- | --- | --- | --- | --- | --- | --- | --- | --- | --- | --- | --- | --- | --- | --- | --- | --- | --- | --- | --- | --- | --- | --- | --- | --- | --- | --- | --- | --- | --- | --- | --- | --- | --- | --- | --- | --- | --- | --- | --- | --- | --- | --- | --- | --- | --- | --- | --- | --- | --- | --- | --- | --- | --- | --- | --- | --- | --- | --- | --- | --- | --- | --- | --- | --- | --- | --- | --- | --- | --- | --- | --- | --- | --- | --- | --- | --- | --- | --- | --- | --- | --- | --- | --- | --- | --- | --- | --- | --- | --- | --- | --- | --- | --- | --- | --- | --- | --- | --- | --- | --- | --- | --- | --- | --- |

**Table S5.** Differential taxa of post therapy and stable phase obtained by Limma analysis

| **Genus** | **logFC** | ***p*-value** | **FDR** |
| --- | --- | --- | --- |
| *Aminicenantales* | -7.518 | 0.0015205 | 0.00583275 |
| *Actinomyces* | 1.168 | 0.00888947 | 0.02476354 |
| *F0332* | 3.029 | 0.0075469 | 0.02153627 |
| *Mobiluncus* | 4.419 | 0.01378603 | 0.03506448 |
| *Mycobacterium* | 5.957 | 0.01541915 | 0.03758417 |
| *Nakamurella* | 5.849 | 4.71E-05 | 0.00039379 |
| *Kineosphaera* | 5.986 | 0.00050756 | 0.00228404 |
| *Rothia* | 1.133 | 0.01349529 | 0.03470218 |
| *Micropruina* | 9.975 | 6.69E-07 | 1.62E-05 |
| *Olsenella* | 7.588 | 4.90E-05 | 0.00039528 |
| *Collinsella* | 3.401 | 0.0095378 | 0.02625701 |
| *Bacteroides* | -3.52 | 6.93E-07 | 1.62E-05 |
| *Phocaeicola* | 3.674 | 0.00484614 | 0.01453841 |
| *Dysgonomonas* | -4.91 | 0.00997612 | 0.02683233 |
| *Prevotellaceae_NK3B31_group* | -7.781 | 0.00054535 | 0.00236318 |
| *Capnocytophaga* | 2.102 | 4.23E-05 | 0.00036683 |
| *Bergeyella* | -2.371 | 0.01757439 | 0.04112407 |
| *Lentimicrobiaceae* | 5.123 | 0.00020073 | 0.00104381 |
| *Prosthecochloris* | 11.361 | 3.25E-07 | 1.15E-05 |
| *Caldithrix* | 7.785 | 4.04E-05 | 0.00036339 |
| *Calditrichaceae* | 6.893 | 9.55E-05 | 0.00069804 |
| *Arcobacter* | 6.101 | 0.01463862 | 0.0363309 |
| *Sulfurovum* | 9.51 | 4.03E-06 | 6.74E-05 |
| *JG30-KF-CM45* | 6.532 | 0.01231448 | 0.03237739 |
| *Chloroplast* | 5.802 | 5.63E-09 | 6.58E-07 |
| *Desulfatiglans* | 6.037 | 0.00013615 | 0.0008384 |
| *Desulfotignum* | 5.554 | 0.00016433 | 0.0009379 |
| *Desulfosarcina* | 8.61 | 1.51E-05 | 0.00016858 |
| *Desulfuromusa* | 8.124 | 2.71E-05 | 0.00026518 |
| *Bacillus* | 7.606 | 3.43E-07 | 1.15E-05 |
| *Solibacillus* | 5.252 | 0.00607308 | 0.01776375 |
| *Sporolactobacillaceae* | 7.058 | 6.11E-07 | 1.62E-05 |
| *Asteroleplasma* | 5.099 | 0.00011004 | 0.00076211 |
| *Catenibacterium* | 6.45 | 0.00391371 | 0.01237579 |
| *UCG-004* | 5.127 | 2.13E-05 | 0.00022688 |
| *Clostridium_innocuum_group* | -2.787 | 0.02232502 | 0.05071898 |
| *Faecalibaculum* | 5.78 | 0.00243462 | 0.00837796 |
| *Ileibacterium* | 9.025 | 2.40E-08 | 1.87E-06 |
| *Abiotrophia* | 2.554 | 8.28E-06 | 0.00012116 |
| *Lactobacillus* | 5.264 | 3.08E-09 | 6.58E-07 |
| *Brochothrix* | 8.857 | 2.69E-06 | 4.86E-05 |
| *Mycoplasma* | 2.219 | 0.04528001 | 0.09056003 |
| *Paenibacillus* | 6.053 | 0.00458361 | 0.01430086 |
| *RF39* | 5.006 | 0.04769179 | 0.09223041 |
| *Ruminiclostridium* | -6.566 | 0.00042654 | 0.00199653 |
| *Clostridium_sensu_stricto_1* | -2.005 | 0.0253403 | 0.05593991 |
| *Clostridium_sensu_stricto_13* | -12.161 | 4.62E-08 | 2.43E-06 |
| *Clostridium_sensu_stricto_18* | -7.187 | 0.00026806 | 0.0013636 |
| *Clostridium_sensu_stricto_7* | -6.895 | 0.00034289 | 0.00167158 |
| *Agathobacter* | -2.672 | 0.04622844 | 0.09167335 |
| *Butyrivibrio* | -1.598 | 0.02518188 | 0.05593991 |
| *Lachnoclostridium* | -2.171 | 0.01067712 | 0.02839143 |
| *Lachnospiraceae_ND3007_group* | -3.398 | 0.03935719 | 0.08296922 |
| *Lachnospiraceae_NK4A136_group* | -5.542 | 0.00375101 | 0.01219077 |
| *Lachnospiraceae_UCG-003* | -8.697 | 1.18E-05 | 0.00015376 |
| *Lachnospiraceae_UCG-004* | -4.546 | 0.00842155 | 0.02374269 |
| *Roseburia* | -2.96 | 0.00472073 | 0.01445441 |
| *Ruminococcus_gnavus_group* | -2.187 | 0.01641862 | 0.0396078 |
| *Butyricicoccus* | -3.816 | 0.00228714 | 0.00805147 |
| *Flavonifractor* | -3.742 | 0.00091583 | 0.00375972 |
| *NK4A214_group* | -6.346 | 0.00046733 | 0.00214424 |
| *Oscillospira* | -4.914 | 0.04720589 | 0.09205149 |
| *Caproiciproducens* | -6.551 | 0.00042661 | 0.00199653 |
| *Faecalibacterium* | -2.16 | 0.00475637 | 0.01445441 |
| *Fournierella* | -4.755 | 0.04829259 | 0.09262678 |
| *Eubacterium_nodatum_group* | -2.049 | 0.00127758 | 0.00498255 |
| *Peptoniphilus* | -3.38 | 0.04676459 | 0.09195726 |
| *Acidaminococcus* | -4.949 | 0.0098593 | 0.02682647 |
| *Anaerovibrio* | -5.737 | 0.0410234 | 0.08420592 |
| *Anaeroglobus* | 2.064 | 0.00726649 | 0.02099207 |
| *Megasphaera* | 1.098 | 0.01474973 | 0.0363309 |
| *Veillonella* | 1.509 | 0.00017139 | 0.00095487 |
| *Methanosaeta* | -7.769 | 0.00120105 | 0.00476348 |
| *Methanolobus* | 5.454 | 0.0055128 | 0.01632906 |
| *LCP-89* | 5.675 | 0.00015557 | 0.00092459 |
| *TM7a* | 7.182 | 0.00027906 | 0.00138934 |
| *TM7x* | -1.756 | 0.00100366 | 0.00404923 |
| *Saccharimonadales* | 1.712 | 0.01812475 | 0.04167434 |
| *Acetobacter* | 4.216 | 0.01345132 | 0.03470218 |
| *Brevundimonas* | 4.889 | 9.15E-06 | 0.00012595 |
| *SWB02* | 5.667 | 0.00057794 | 0.00244962 |
| *Rhodobacter* | 8.027 | 0.00011073 | 0.00076211 |
| *Mitochondria* | 6.936 | 2.70E-06 | 4.86E-05 |
| *Sphingopyxis* | 5.139 | 0.01816574 | 0.04167434 |
| *Marinobacter* | 7.569 | 0.00019541 | 0.0010392 |
| *Bordetella* | -11.366 | 5.41E-06 | 8.44E-05 |
| *Cupriavidus* | 6.08 | 0.01450683 | 0.0363309 |
| *Lautropia* | 2.197 | 0.00013565 | 0.0008384 |
| *Comamonas* | 2.036 | 0.01729701 | 0.04088384 |
| *Kingella* | -5.654 | 1.32E-05 | 0.00016214 |
| *Ellin6067* | 5.398 | 0.00188303 | 0.0069941 |
| *Dechloromonas* | 5.671 | 0.01668156 | 0.03983148 |
| *Denitromonas* | 4.827 | 0.0021369 | 0.00781303 |
| *AAP99* | 6.657 | 0.00012202 | 0.00081577 |
| *Sutterella* | -6.332 | 0.00018231 | 0.00099213 |
| *T34* | -5.668 | 0.04182334 | 0.0851014 |
| *Cardiobacterium* | 1.953 | 0.00276508 | 0.00924327 |
| *Candidatus_Thiobios* | 7.299 | 6.59E-05 | 0.00049746 |
| *Thiorhodococcus* | 8.129 | 2.72E-05 | 0.00026518 |
| *Candidatus_Thiodiazotropha* | 6.111 | 0.00013158 | 0.0008384 |
| *Sedimenticola* | 7.457 | 5.70E-05 | 0.0004443 |
| *Candidatus_Competibacter* | 10.061 | 5.19E-08 | 2.43E-06 |
| *Klebsiella* | 3.407 | 0.00385493 | 0.01235691 |
| *Mangrovibacter* | -9.222 | 3.15E-05 | 0.00029504 |
| *Plesiomonas* | 7.392 | 0.0022973 | 0.00805147 |
| *Proteus* | -8.535 | 0.00266742 | 0.00904602 |
| *Serratia* | 2.509 | 0.02807873 | 0.06083724 |
| *Actinobacillus* | -3.126 | 0.00230534 | 0.00805147 |
| *Aggregatibacter* | -2.085 | 0.00187881 | 0.0069941 |
| *Acinetobacter* | 3.665 | 0.03713655 | 0.07972434 |
| *Pseudomonas* | 7.482 | 1.45E-05 | 0.00016858 |
| *Thiothrix* | 5.668 | 0.00015805 | 0.00092459 |
| *Photobacterium* | 8.001 | 0.003345 | 0.01102436 |
| *Vibrio* | 5.554 | 1.51E-06 | 3.21E-05 |
| *Ahniella* | 5.864 | 0.000533 | 0.00235326 |
| *Dokdonella* | 5.28 | 0.0399897 | 0.08325179 |
| *Sphaerochaeta* | 3.132 | 0.02355565 | 0.0530002 |
| *Zixibacteria* | 5.307 | 0.00058623 | 0.00244962 |

**Table S6.** The discriminating taxon features among the three disease states across different taxonomic levels

| **Taxa** | **Group** | **LDA** | **FDR *p*-value** |
| --- | --- | --- | --- |
| *Pseudomonas* | acute | 4.05 | 1.01E-06 |
| *Haemophilus* | acute | 4.01 | 0.002323 |
| *Veillonella* | post therapy | 3.69 | 0.001083 |
| *Lautropia* | post therapy | 3.55 | 0.000941 |
| *Actinobacillus* | Stable | 3.53 | 0.001166 |
| *Neisseria* | acute | 3.44 | 0.013246 |
| *Corynebacterium* | acute | 3.32 | 0.003148 |
| *Rothia* | post therapy | 3.31 | 0.004675 |
| *Klebsiella* | acute | 3.12 | 0.001911 |
| *Gemella* | acute | 2.94 | 0.000214 |
| *Moraxella* | acute | 2.63 | 1.01E-06 |
| *Aggregatibacter* | acute | 2.63 | 7.99E-05 |
| *Fusobacterium* | acute | 2.62 | 0.059192 |
| *Clostridium_sensu_stricto_13* | stable | 2.41 | 0.000527 |

**Table S7.** Correlation between taxa and clinical features

| **Genus** | **Clinical features** | **R** | **FDR *p*-value** |
| --- | --- | --- | --- |
| ***Capnocytophaga*** | EO count | -0.48184 | 0.00942 |
| ***Klebsiella*** | EO count | 0.40273 | 0.0336 |
| ***Capnocytophaga*** | EO percentage | -0.41514 | 0.02804 |
| ***Klebsiella*** | EO percentage | 0.41201 | 0.02937 |
| ***Actinomyces*** | FEV_1_ / pre | 0.39229 | 0.03894 |
| ***Leptotrichia*** | IL-23 | 0.44142 | 0.0187 |
| ***Leptotrichia*** | IL-4 | 0.61987 | 0.00043 |
| ***Veillonella*** | IL-4 | 0.61295 | 0.00052 |
| ***Actinomyces*** | IL-4 | 0.48789 | 0.00844 |
| ***Neisseria*** | IL-4 | 0.43846 | 0.0196 |
| ***Rothia*** | IL-4 | 0.42758 | 0.02323 |
| ***Neisseria*** | IL-5 | 0.58824 | 0.00099 |
| ***Rothia*** | IL-5 | 0.47108 | 0.0114 |
| ***Porphyromonas*** | IL-5 | 0.46911 | 0.01179 |
| ***Corynebacterium*** | PCT | 0.44104 | 0.01881 |
| ***Fusobacterium*** | TNF-α | -0.65217 | 0.00017 |
| ***Pseudomonas*** | TNF-α | 0.50544 | 0.00608 |
| ***Prevotella*** | TNF-α | -0.48814 | 0.00841 |
| ***Leptotrichia*** | TNF-α | -0.48715 | 0.00856 |
| ***Gemella*** | TNF-α | -0.43182 | 0.02176 |
| ***Rothia*** | TNF-α | -0.42688 | 0.02348 |
| ***Porphyromonas*** | TNF-α | -0.41996 | 0.02609 |
| ***Porphyromonas*** | TTV load | -0.62549 | 0.00037 |
| ***Klebsiella*** | TTV load | 0.45161 | 0.01585 |
| ***Actinobacillus*** | TTV load | -0.42984 | 0.02243 |

**Abbreviations:** EO, eosinophils; FEV_1_, forced expiratory volume in 1 s; IL, interleukin; PCT, procalcitonin; TNF, tumor necrosis factor; TTV, Torque Teno virus.

**Table S8.** Relative abundance of BugBase annotation phenotypes

| **Phenotypes（%）** | **Acute** | **Post-therapy** | **Stable** | ***p-*value** |
| --- | --- | --- | --- | --- |
| Gram Positive | 39.0 (18.4-63.3) | 39.5 (24.6-62.4) | 37.0 (30.8-63.1) | 0.726 |
| Gram Negative | 61.0 (36.6-81.6) | 60.5 (37.6-75.4) | 63.0 (36.9-69.2) | 0.726 |
| Potentially Pathogenic | 27.0 (16.7-51.1) | 18.6 (8.4-42.2) | 20.6 (4.6-46.9) | 0.153 |
| Forms Biofilms ^a^ | 48.3 ± 20.4 | 44.5 ± 22.3 | 36.4 ± 20.1 | 0.082 |
| Contains Mobile Elements | 25.2 (9.4-50.7) | 16.7 (11.2-30.3) | 19.3 (12.3-47.2) | 0.732 |
| Oxygen Utilizing |  |  |  |  |
| Aerobic | 25.6 (19.3-46.1) | 36.0 (17.5-46.9) | 26.0 (15.6-41.0) | 0.455 |
| Anaerobic | 25.2 (13.9-35.0) | 29.9 (23.4-43.4) | 38.7 (17.8-50.5) | 0.105 |
| Facultatively Anaerobic | 27.0 (15.8-53.9) | 22.9 (12.0-31.7) | 16.1 (11.9-49.8) | 0.206 |
| Oxidative Stress Tolerant | 27.0 (16.8-51.2) | 18.6 (8.5-42.6) | 20.7 (4.6-46.6) | 0.144 |

**Table S9.** Composition of the clusters

| Cluster | Acute（n） | Post-therapy（n） | Stable（n） |
| --- | --- | --- | --- |
| MClus1 | 25 | 20 | 1 |
| MClus2 | 2 | 8 | 0 |
| MClus3 | 4 | 1 | 25 |
| MClus4 | 0 | 4 | 10 |

**Table S10.** Smoking status of the clusters

| Cluster | Never smoked (n) | Quit smoking (n) | Current smoking (n) |
| --- | --- | --- | --- |
| MClus1 | 11 | 20 | 15 |
| MClus2 | 1 | 8 | 1 |
| MClus3 | 8 | 14 | 8 |
| MClus4 | 5 | 6 | 3 |

**Table S11.** Alpha diversity index of clusters

| **Alpha diversity** | **hclust1** | **hclust2** | **hclust3** | **hclust4** | ***p*** |
| --- | --- | --- | --- | --- | --- |
| **ACE** | 306.9 ± 105.0 | 498.6 ± 108.3 | 333.8 ± 62.6 | 275.3 ± 141.5 | < 0.0001 |
| **Shannon** | 3.4 (2.6-3.9) | 4.2 (3.9-4.5) | 3.8 (3.1-4.3) | 3.4 (2.1-3.8) | 0.005 |

**Table S12**. Levels of cytokines in clusters

| Cytokine  (pg/ml) | MClus1 | MClus2 | MClus3 | MClus4 | *p*-value |
| --- | --- | --- | --- | --- | --- |
| TNF-α | 41.0 (18.2-101.0) | 21.3 (12.8-42.8) | 21.9 (13.5-40.1) | 21.9 (11.2-123.7) | 0.383 |
| IL-6 | 63.2 (14.1-263.9) | 44.5 (25.8-195.1) | 40.2 (24.8-123.7) | 344.6 (36.7-1386.2) | 0.279 |
| IL-25 | 321.2 (132.5-580.2) | 406.2 (244.1-698.2) | 364.8 (248.1-507.3) | 220.5 (180.2-319.8) | 0.201 |
| IL-4 | 45.9 (21.2-87.2) | 70.0 (41.2-119.7) | 76.0 (63.3-93.2) | 53.4 (39.4-63.0) | 0.057 |
| IL-17A | 18.6 (7.2-32.1) | 17.1 (9.9-41.4) | 27.8 (18.6-38.5) | 14.6 (11.3-22.7) | 0.062 |
| IL-5 | 7.0 (3.3-19.3) | 17.7 (9.3-27.2) | 19.4 (15.4-26.2) | 11.9 (5.1-21.5) | 0.007 |
| IL-23 | 234.6 (146.4-390.4) | 236.4 (145.6-542.5) | 339.9 (279.4-462.6) | 309.0 (256.7-539.1) | 0.064 |

**Reference**

1. Yu Y, Zhao L, Xie Y, et al. Th1/Th17 Cytokine Profiles are Associated with Disease Severity and Exacerbation Frequency in COPD Patients. *Int J Chron Obstruct Pulmon Dis* 2020; **15**: 1287-99.

2. Salter SJ, Cox MJ, Turek EM, et al. Reagent and laboratory contamination can critically impact sequence-based microbiome analyses. *BMC Biol* 2014; **12**: 87.

3. Xie Y, Xue Q, Jiao W, et al. Associations Between Sputum Torque Teno Virus Load and Lung Function and Disease Severity in Patients With Chronic Obstructive Pulmonary Disease. *Front Med (Lausanne)* 2021; **8**: 618757.
